# The value of bile acid spectrum in the evaluation of hepatic injury in children with infectious mononucleosis caused by Epstein Barr virus infection

**DOI:** 10.3389/fped.2023.1109762

**Published:** 2023-03-21

**Authors:** Ren Shen, Yan Zhou, Lintao Zhang, Shanpu Yang

**Affiliations:** Department of Pediatrics, Yuhuan People's Hospital, Taizhou, China

**Keywords:** epstein-Barr virus, infectious mononucleosis, bile acid, hepatic injury, lymphocyte subsets

## Abstract

**Background:**

Infectious mononucleosis (IM) is an acute infectious disease, caused by Epstein-Barr virus (EBV) infection, which can invade various systemic systems, among which hepatic injury is the most common. In this study, ultra performance liquid chromatography-tandem mass spectrometry (UPLC-MS/MS) was used to detect serum bile acid spectrum in IM children quantitatively, and to investigate its role in the early assessment of hepatic injury.

**Methods:**

This case-control study was conducted at Yuhuan People's Hospital. A total of 60 IM children and 30 healthy children were included in the study. Among 60 children with IM, 30 had hepatic injury, and 30 without hepatic injury. The clinical and laboratory data were analyzed, and the serum bile acid spectrum and lymphocyte subsets were evaluated in the three groups.

**Results:**

There were statistically significant differences in cholic acid (CA), chenodeoxycholic acid (CDCA), deoxycholic acid (DCA), lithocholic acid (LCA), glycochenodeoxycholic acid (GCDCA), glycodeoxycholic acid(GDCA), glycolithocholic acid (GLCA), taurocholic acid (TCA), taurochenodeoxycholic acid (TCDCA), taurodeoxycholic acid (TDCA), ursodeoxycholic acid (UDCA), glycoursodeoxycholic acid (GUDCA), tauroursodeoxycholic acid(TUDCA), percentage of NK cells, CD4+ and CD8+ in IM hepatic injury group, without hepatic injury group, and the healthy control group (*P* < 0.05). The percentage of NK cells was positively correlated with TCA (*P* < 0.05); it was negatively correlated with CDCA, DCA, LCA, GCDCA, GDCA, GLCA, TDCA, UDCA, GUDCA, TUDCA (*P* < 0.05). CD4+ was positively correlated with CA, TCA and TCDCA (*P* < 0.05); it was negatively correlated with CDCA, DCA, LCA, GCDCA, GDCA, GLCA, TDCA, UDCA, GUDCA and TUDCA (*P* < 0.05). CD8+ was positively correlated with CDCA, DCA, LCA, GCDCA, GDCA, GLCA, TDCA, UDCA, GUDCA and TUDCA (*P* < 0.05); it was negatively correlated with CA, TCA and TCDCA (*P* < 0.05). ROC curve analysis showed that CD8+, GDCA and GLCA had high predictive value for hepatic injury in IM patients.

**Conclusions:**

UPLC-MS/MS method can sensitively detect the changes in serum bile acid spectrum before hepatic injury in children with IM, which is helpful for early assessment of hepatic injury in children with IM. The changes in lymphocyte subsets in IM children are related to some bile acid subfractions, which may be related to IM hepatic injury.

## 1. Introduction

IM is an acute infectious disease caused by EBV, mainly affecting children and adolescents. It is clinically characterized by fever, angina, hepatosplenomegaly, and lymphadenopathy (1, 2). In Europe and the United States, IM mainly affects adolescents and adults aged 10–30 years (3). In China, IM tends to occur in children aged 4–6 (4, 5). The hepatic injury is one of the most common manifestations of IM. Studies have shown that up to 80%–90% of patients have mild or moderate hepatic injury, most of which is temporary, and a few may have severe hepatitis or liver failure (6–8). Bile acid is the main component of bile, the general name of a class of hydroxyl derivatives of 24-carbon cholestane acid. It belongs to endogenous organic anions. The enterohepatic circulation of bile acid belongs to “closed” circulation. Although the bile acid concentration in portal vein blood is very high, the content of bile acid entering the systemic circulation is usually shallow. When the liver is slightly damaged, the amount of bile acid entering the peripheral blood increases and the composition changes. The quantitative detection of the bile acid spectrum plays an important role in assessing liver disease (9, 10). There are few reports on the relationship between the bile acid spectrum and hepatic injury in children with infectious mononucleosis. In this study, total bile acid (TBA) and 15 bile acid subfractions in the serum of IM children were quantitatively detected by UPLC-MS/MS to explore their role in the early assessment of hepatic injury.

## 2. Materials and methods

### 2.1. Patient characteristics

Inpatients who were hospitalized from September 1, 2020 to July 31, 2022, were selected from the case system of Yuhuan People's Hospital. All patients' legal guardian have signed written informed consent at admission. This research scheme was reviewed and approved by the Ethics Committee of Yuhuan People's Hospital (No: 2020–005). All procedures performed in this study involving human participants were following the Declaration of Helsinki. A total of 60 IM children and 30 healthy children were included in the study. According to alanine aminotransferase level, 60 children with IM were divided into hepatic injury group (30 cases) and without hepatic injury group (30 cases); In addition, 30 children who need blood sampling for physical examination in primary health care were taken as healthy control group, and the number of blood sampling was not increased.

Inclusion criteria: (1) Meet the IM diagnostic criteria (11, 12): meet at least three clinical manifestations as follows: (a) fever, (b) angina, (c) cervical lymph node enlargement, (d) liver enlargement, (e) spleen enlargement, (f) eyelid edema; and the laboratory evidence of any primary EBV infection: (a) anti-EBV-capsid antigen-IgM and anti-EBV-capsid antigen-IgG antibodies are positive, and anti-EBV-nuclear antigen-IgG is negative; (b) single anti-EBV-capsid antigen-IgG antibody is positive, and EBV-capsid antigen-IgG is a low-affinity antibody. All subjects selected in this study met the above diagnostic criteria. (2) Ages 1–14, Han nationality. (3) Volunteer to join the study and sign the informed consent.

Exclusion criteria: (1) With other severe underlying systemic or hepatobiliary diseases, including autoimmune diseases, hepatitis, gallstones, and drug-induced liver disease. (2) With other serious infectious diseases, incluiding acute suppurative tonsillitis, sepsis, AIDS, cytomegalovirus infection, adenovirus infection, and human herpesvirus 6 infection.

### 2.2. Liver function test

Blood samples were collected by experienced nurses when the subjects were fasting. Aptio automatic biochemical assembly line is used to detect the indexes of alanine aminotransferase and glutamic oxaloacetic aminotransferase. The methodology is the rate method. In this study, ALT ≥ 1 ULN (1 ULN: 40 U/L) was defined as combined hepatic injury according to previous literature (13).

### 2.3. Bile acid spectrum detection

Blood samples were collected by experienced nurses when the subjects were fasting. The serum bile acid spectrum was determined by UPLC-MS/MS, which Hangzhou Hanku Medical Laboratory Co., LTD completed.

### 2.4. Detection of T lymphocyte subsets

The percentage of NK cells, helper T cells (CD4+), killer T cells (CD8+), and CD4+/CD8+ values were detected by flow cytometry, which Hangzhou Adicom Medical Laboratory Center performed.

### 2.5. EBV-specific antibody detection

Serum EBV-specific antibodies were detected by direct chemiluminescence two-step indirect immunoassay, which Hangzhou Adicom Medical Laboratory Center conducted.

### 2.6. Statistical analysis

Shapiro–Wilk normality test is used to determine whether the data is normally distributed. Values are expressed as mean ± standard deviation or median and quartile ranges. Mann–Whitty *U* test and Kruskal–Wallis test were used for data with the non-normal distribution. The normal distribution variables were analyzed by *t*-test and ANOVA. Spearman correlation analysis was used for hierarchical variable data and Pearson correlation analysis was used for continuous variable data. All statistical analyses were performed with SPSS version 23.0, and the difference was considered statistically significant with *P* < 0.05.

## 3. Results

### 3.1. Clinical and experimental characteristics of IM hepatic injury group, without hepatic injury group and healthy control group

30 patients in IM hepatic injury group, including 13 males and 17 females, with an average age of 5.0 ± 1.7 years, the minimum age of 1.7 years, the maximum age of 8.3 years, the course of disease 2.8 ± 0.9 days, the shortest course of disease 1.0 days, and the longest duration of disease 4.0 days. 30 patients without hepatic injury group in IM group, including 14 males and 16 females, with an average age of 5.5 ± 1.8 years, the minimum age of 3.3 years, the maximum age of 10.3 years, the course of disease 2.4 ± 0.9 days, the shortest course of disease 1.0 days, and the longest course of disease 4.0 days. There were 30 healthy controls, including 16 males and 14 females, with an average age of 5.7 ± 2.0 years, the minimum age of 1.5 years and the maximum age of 11.3 years. There was no significant difference in sex and age between IM hepatic injury group, without hepatic injury group, and the healthy control group (*P* > 0.05). There was no significant difference in the disease course between IM hepatic injury group and without hepatic injury group (*P* > 0.05). There were statistically significant differences in CA, CDCA, DCA, LCA, GCDCA, GDCA, GLCA, TCA, TCDCA, TDCA, UDCA, GUDCA, TUDCA, CA/CDCA, percentage of NK cells, CD4+/CD8+, CD4+ and CD8+ in IM hepatic injury group, without hepatic injury group and healthy control group (*P *< 0.05). At the same time, there were no significant differences in GCA, TLCA and TBA (*P *> 0.05) (Table 1).

**Table 1:** Comparison of laboratory findings between IM hepatic injury group, without hepatic injury and healthy control group.

Characteristic	IM hepatic injury group (*n* = 30)	IM without hepatic injury group (*n* = 30)	Healthy control group (*n* = 30)	*P*
Sex (male)	13 (43.3)	14 (46.7)	16 (53.3)	0.732
Age (years)	5.0 ± 1.7 (1.7–8.3)	5.5 ± 1.8 (3.3–10.3)	5.7 ± 2.0 (1.5–11.3)	0.362
**Disease course (days)**	2.8 ± 0.9 (1.0–4.0)	2.4 ± 0.9 (1.0–4.0)	–	0.125
CA (nmol/L)	33.2 (19.3–113.0)	31.9 (19.7–76.7)	67.9 (40.9–128.8)	0.030
CDCA (nmol/L)	140.6 (65.8–369.9)	202.6 (93.5–398.2)	38.2 (28.5–82.2)	<0.001
DCA (nmol/L)	57.1 (12.3–120.1)	80.7 (12.5–169.0)	5.5 (5.1–5.8)	<0.001
LCA (nmol/L)	9.9 (5.7–15.4)	6.2 (3.5–20.4)	1.6 (0.7–2.8)	<0.001
GCA (nmol/L)	544.1 (319.7–1365.6)	447.7 (305.0–909.6)	809.7 (470.1–1523.7)	0.109
GCDCA (nmol/L)	2173.1 (1234.9–4717.8)	1561.4 (928.5–2490.9)	636.2 (319.7–785.6)	<0.001
GDCA (nmol/L)	155.3 (16.8–359.0)	87.5 (27.2–244.4)	0.9 (0.3–1.1)	<0.001
GLCA (nmol/L)	3.0 (1.3–9.3)	2.1 (0.8–5.9)	0.5 (0.4–0.7)	<0.001
TCA (nmol/L)	189.6 (99.9–366.1)	135.4 (45.9–257.7)	807.9 (580.8–1625.3)	<0.001
TCDCA (nmol/L)	668.5 (304.4–1531.4)	494.3 (201.6–877.7)	1407.5 (808.4–1917.6)	<0.001
TDCA (nmol/L)	57.4 (3.6–118.7)	23.4 (9.3–76.3)	0.3 (0.2–0.5)	<0.001
TLCA (nmol/L)	1.6 (0.6–4.0)	1.1 (0.5–2.4)	1.3 (1.0–1.6)	0.461
UDCA (nmol/L)	41.2 (12.7–99.5)	49.4 (30.1–153.2)	4.0 (2.3–6.8)	<0.001
GUDCA (nmol/L)	204.5 (128.8–293.1)	182.9 (87.7–351.4)	26.6 (10.4–34.6)	<0.001
TUDCA (nmol/L)	26.7 (10.9–43.0)	21.9 (9.5–45.6)	0.8 (0.4–1.7)	<0.001
CA/CDCA	0.3 (0.2–0.4)	0.2 (0.1–0.5)	1.6 (1.2–2.4)	<0.001
TBA (µmol/L)	5.5 (3.5–10.8)	4.0 (2.2–5.4)	4.2 (2.9–6.1)	0.171
NK cell (%)	5.1 (3.9–7.2)	6.9 (5.1–10.0)	11.3 (9.8–13.7)	<0.001
CD4+/CD8+ ratio	0.2 (0.1–0.3)	0.3 (0.2–0.4)	1.2 (1.0–1.5)	<0.001
CD4+ (%)	13.2 (10.3–18.3)	17.2 (12.5–20.6)	39.4 (33.6–42.2)	<0.001
CD8+ (%)	65.0 (55.8–74.2)	58.3 (47.6–64.3)	32.5 (27.9–35.3)	<0.001

The data are expressed in median (quartile range), mean ± standard deviation and *n* (%); *T*-test and ANOVA were used for normal distribution variables, and chi square test was used for categorical variables; Kruskal–Wallis test is used for data with non normal distribution.

### 3.2. Correlation between 15 bile acid subfractions and T lymphocyte subsets

The percentage of NK cells was positively correlated with TCA and CA/CDCA (*P* < 0.05); it was negatively correlated with CDCA, DCA, LCA, GCDCA, GDCA, GLCA, TDCA, UDCA, GUDCA, TUDCA (*P* < 0.05). CD4+/CD8+ was positively correlated with CA, TCA, TCDCA and CA/CDCA (*P* < 0.05); it was negatively correlated with CDCA, DCA, LCA, GCDCA, GDCA, GLCA, TDCA, UDCA, GUDCA, TUDCA (*P* < 0.05). CD4+ was positively correlated with CA, TCA, TCDCA and CA/CDCA (*P* < 0.05); it was negatively correlated with CDCA, DCA, LCA, GCDCA, GDCA, GLCA, TDCA, UDCA, GUDCA and TUDCA (*P* < 0.05). CD8+ was positively correlated with CDCA, DCA, LCA, GCDCA, GDCA, GLCA, TDCA, UDCA, GUDCA and TUDCA (*P* < 0.05); it was negatively correlated with CA, TCA, TCDCA and CA/CDCA (*P* < 0.05) (Table 2).

**Table 2:** Correlation between bile acid spectrum and T lymphocyte subsets.

	NK cell (%)		CD4+/CD8+		CD4+		CD8+	
Bile acid spectrum	*r*	*P*	*r*	*P*	*r*	*P*	*r*	*P*
CA (nmol/L)	0.197	0.062	0.316	0.002	0.289	0.006	−0.364	<0.001
CDCA (nmol/L)	−0.268	0.011	−0.355	0.001	−0.392	<0.001	0.285	0.007
DCA (nmol/L)	−0.425	<0.001	−0.515	<0.001	−0.549	<0.001	0.457	<0.001
LCA (nmol/L)	−0.236	0.025	−0.597	<0.001	−0.614	<0.001	0.515	<0.001
GCA (nmol/L)	0.204	0.054	0.169	0.111	0.177	0.095	−0.125	0.239
GCDCA (nmol/L)	−0.266	0.011	−0.445	<0.001	−0.463	<0.001	0.417	<0.001
GDCA (nmol/L)	−0.509	<0.001	−0.646	<0.001	−0.678	<0.001	0.593	<0.001
GLCA (nmol/L)	−0.409	<0.001	−0.575	<0.001	−0.616	<0.001	0.474	<0.001
TCA (nmol/L)	0.416	<0.001	0.590	<0.001	0.605	<0.001	−0.513	<0.001
TCDCA (nmol/L)	0.186	0.080	0.383	<0.001	0.380	<0.001	−0.335	0.001
TDCA (nmol/L)	−0.540	<0.001	−0.648	<0.001	−0.680	<0.001	0.592	<0.001
TLCA (nmol/L)	−0.081	0.447	−0.072	0.497	−0.087	0.413	0.026	0.808
UDCA (nmol/L)	−0.336	0.001	−0.392	<0.001	−0.433	<0.001	0.312	0.003
GUDCA (nmol/L)	−0.437	<0.001	−0.506	<0.001	−0.523	<0.001	0.479	<0.001
TUDCA (nmol/L)	−0.464	<0.001	−0.530	<0.001	−0.547	<0.001	0.500	<0.001
CA/CDCA	0.442	<0.001	0.588	<0.001	0.590	<0.001	−0.577	<0.001
TBA (µmol/L)	−0.043	0.686	−0.022	0.835	−0.037	0.730	0.030	0.779

Spearman correlation analysis was used.

### 3.3. Predictive value of Cd8+ and bile acid spectrum subcomponents on hepatic injury in patients with IM

**Figure 1:**
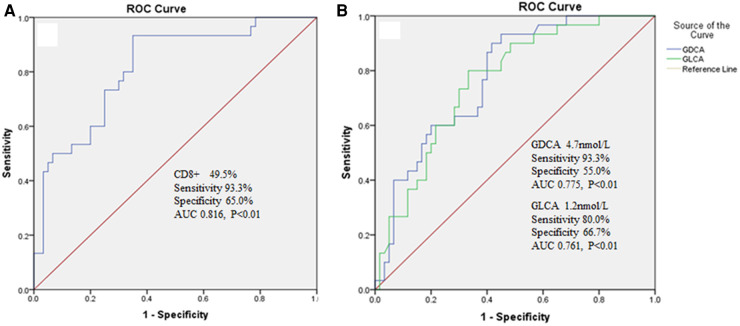
(**A**) The predictive value of CD8+ on hepatic injury in IM patients. (**B**) The predictive value of GDCA and GLCA on hepatic injury in IM patients.

ROC curve was used to evaluate the predictive value of CD8+, GDCA and GLCA for hepatic injury in IM patients. When the cut-off value for CD8+ was set at 49.5%, the sensitivity was 93.3%, the specificity was 65.0% with an area under the curve calculated to be 0.816 (Figure 1A). When the cut-off value for GDCA was set at 4.7 nmol/L, the sensitivity was 93.3%, the specificity was 55.0% with an area under the curve calculated to be 0.775 (Figure 1B). When the cut-off value for GLCA was set at 1.2 nmol/L, the sensitivity was 80.0%, the specificity was 66.7% with an area under the curve calculated to be 0.761 (Figure 1B).

## 4. Discussion

IM is an acute infectious diseases caused by EBV infection, which can invade all body systems, among which hepatic injury is the most common (14). Most children suffer from mild to moderate hepatic injury, and the liver function returns to normal within the first few months(6, 8, 15). In a small number of patients with chronic active EBV infection, hepatic injury can show an irreversible chronic development process, leading to cirrhosis and even liver failure (16–19). However, this is outcome is absolutely exceptional in the immunocompetent child. At present, the pathophysiological mechanism of how EBV infection leads to hepatic injury has not been fully clarified.

Some researchers believe that after EBV infects cells, it will lead to the free radicals generated by lipid peroxidation, which will produce toxic effects and lead to liver cell damage. At the same time, EBV itself has no direct cytotoxic effect on liver cells (20). It has also been reported that EBV does not infect liver cells, bile duct epithelial cells or vascular endothelial cells, but the infiltration of CD8+ T cells indirectly leads to hepatic injury (21). In the acute phase of IM, the number of CD4+ regulatory T cells decreased significantly, and the immunosuppressive function of the body decreased, which contributed to the activation and proliferation of effector T cells and CD8+ T cells and the release of inflammatory cytokines, and promoted the host immune system to clear EBV. In the recovery period of IM, CD4+ regulates the number of T cells to return to normal, and the immune suppression function of the body is enhanced, which can inhibit the excessive proliferation of effector T cells and the release of inflammatory cytokines and avoid the extreme immune damage of the body (22, 23). In this study, it was found that there were statistically significant differences in the percentage of NK cells, CD4+, CD8+ and CD4+/CD8+ values between IM hepatic injury group, without hepatic injury group and the healthy control group. ROC curve analysis showed that CD8+ had a high predictive value for liver injury in IM patients. The results of this study are similar to those of previous studies (21–23). However, how do these changes in lymphocyte subsets affect liver function? This study found that the percentage of NK cells was positively correlated with TCA and CA/CDCA, and negatively correlated with CDCA, DCA, LCA, GCDCA, GDCA, GLCA, TDCA, UDCA, GUDCA, TUDCA. The CD4+/CD8+ was positively correlated with CA, TCA, TCDCA and CA/CDCA; it was negatively correlated with CDCA, DCA, LCA, GCDCA, GDCA, GLCA, TDCA, UDCA, GUDCA, TUDCA. These results suggest that the changes in lymphocyte subsets in IM patients may lead to changes in the composition of bile acids *in vivo*. It has been found that bile acid not only plays an important role in the digestion and absorption of lipids and the steady metabolism of cholesterol, but also plays an important regulatory role in metabolism and liver regeneration as a signal molecule (24). The hepatic injury of IM patients is likely to be caused by the influence of immune cell changes on the secretion and metabolism of bile acid subfractions, thus leading to the damage of liver cells.

Research shows that the order of hydrophilicity of common free and conjugated bile acids in the human body is: UDCA > CA > CDCA > DCA > LCA, taurine conjugated bile acid > glycine conjugated bile acid > free bile acid (25). The contents of hydrophobic and hepatotoxic bile acids in the bile acid spectrum of patients with liver cirrhosis increased significantly, which may lead to changes in the bacterial flora in the bile, thus transforming primary bile acids into secondary bile acids, thus leading to a vicious cycle (26). In this study, ROC curve analysis shows that GDCA and GLCA have a high predictive value for hepatic injury in IM patients, which may be related to the low hydrophilicity and high hepatotoxicity of GDCA and GLCA, which may be a new clue for the development of new liver function evaluation indicators in the future. In addition, many foreign studies have shown that in the process of a pathological state, the content of total bile acid and some hydrophobic bile acids in the liver increased significantly. Through molecular biology experiments, it was found that the transcriptional level expression of bile acid synthase CYP7A1 and some transporter genes BSEP, ASBT, NTCP, FXR, FGF15 decreased significantly, further supporting the possible change rule of the bile acid spectrum (9, 27). These results indicate that the changes in bile acid spectrum are consistent with the changes in clinical indicators, suggesting that bile acid plays an important role in assessing liver disease. The results of this study showed that there were significant differences in CA, CDCA, DCA, LCA, GCDCA, GDCA, GLCA, TCA, TCDCA, TDCA, UDCA, GUDCA, TUDCA, CA/CDCA between IM hepatic injury, without hepatic injury group and the healthy control group. We found that the bile acid composition of IM patients with normal liver function changed significantly compared with the healthy control group, suggesting that bile acid spectrum detection is more sensitive than routine liver function examination to detect potential damage to liver function at an early stage, which is convenient for clinicians to timely assess the condition.

This study has several deficiencies, which need to be improved in the future. First, bile acid spectrum and lymphocyte subsets were detected only in the acute phase of IM, and the sample size of the study was too small. Secondly, in the future, we can further study the evaluation value of bile acid spectrum on liver function after other human herpes virus infections, and further explore the change characteristics of bile acid spectrum in different herpes virus infections.

## 5. Conclusions

UPLC-MS/MS method can sensitively detect the changes in serum bile acid spectrum before liver function damage in children with IM, which is helpful for early assessment of hepatic injury in children with IM. The changes of lymphocyte subsets in children with IM were correlated with some bile acid subfractions, which provided a new idea for studying the mechanism of hepatic injury in children with IM in the future.

## Data Availability

The original contributions presented in the study are included in the article/Supplementary Material, further inquiries can be directed to the corresponding author.
